# Inhaled Corticosteroids and the Pneumonia Risk in Patients With Chronic Obstructive Pulmonary Disease: A Meta-analysis of Randomized Controlled Trials

**DOI:** 10.3389/fphar.2021.691621

**Published:** 2021-06-29

**Authors:** Hong Chen, Jian Sun, Qiang Huang, Yongqi Liu, Mengxin Yuan, Chunlan Ma, Hao Yan

**Affiliations:** ^1^ Department of Respiratory and Critical Care Medicine, Chengdu Second People’s Hospital, Chengdu, China; ^2^ Department of Respiratory, The First Affiliated Hospital of Chengdu Medical College, Chengdu, China

**Keywords:** inhaled corticosteroids, chronic obstructive pulmonary disease, adverse event, pneumonia, meta-analysis

## Abstract

**Background:** Whether all types of inhaled corticosteroids (ICSs) would increase the pneumonia risk in patients with chronic obstructive pulmonary disease (COPD) remains controversial. We aimed to assess the association between ICSs treatment and pneumonia risk in COPD patients, and the impact of medication details and baseline characteristics of patients on the association.

**Methods:** Four databases (PubMed, Embase, Cochrane Library, and Clinical Trials.gov) were searched to identify eligible randomized controlled trials (RCTs) comparing ICSs treatment with non-ICSs treatment on the pneumonia risk in COPD patients. Pooled results were calculated using Peto odds ratios (Peto ORs) with corresponding 95% confidence intervals (CIs).

**Results:** A total of 59 RCTs enrolling 103,477 patients were analyzed. All types of ICSs significantly increased the pneumonia risk (Peto OR, 1.43; 95% CI, 1.34–1.53). Subgroup analysis showed that there was a dose-response relationship between ICSs treatment and pneumonia risk (low-dose: Peto OR, 1.33; 95% CI, 1.22–1.45; medium-dose: Peto OR, 1.50; 95% CI, 1.28–1.76; and high-dose: Peto OR, 1.64; 95% CI, 1.45–1.85). Subgroup analyses based on treatment durations and baseline characteristics (severity, age, and body mass index) of patients were consistant with the above results. Subgroup analysis based on severity of pneumonia showed that fluticasone (Peto OR, 1.75; 95% CI, 1.44–2.14) increased the risk of serious pneumonia, while budesonide and beclomethasone did not.

**Conclusions:** ICSs treatment significantly increased the risk of pneumonia in COPD patients. There was a dose-response relationship between ICSs treatment and pneumonia risk. The pneumonia risk was related with COPD severity.

## Introduction

Chronic obstructive pulmonary disease (COPD) is currently the third leading cause of death in the world, and acute exacerbations contribute substantially to this (GBD 2015 Chronic Respiratory Disease Collaborators, 2017; Viniol and Vogelmeier, 2018; López-Campos et al., 2019). Treatment and prevention of repeated exacerbations have been identified as a priority by the Global Initiative for Chronic Obstructive Lung Disease (GOLD). Currently, the management of patients with stable COPD mainly relies on inhaled agents such as inhaled corticosteroids (ICSs), long-acting muscarinic antagonist (LAMA), long-acting β-agonist (LABA), etc. Among them, ICSs have been recommended by GOLD as first-line maintenance treatment in patients with repeated exacerbations to relieve the frequency and severity of acute exacerbations of COPD, and improve their quality of life (Yang et al., 2017).

Some recent studies have raised concerns about increased pneumonia risk associated with long-term use of ICSs (Dong et al., 2014; Kew and Seniukovich, 2014; Morjaria et al., 2017; Janson et al., 2018; Yang et al., 2019; Zhang et al., 2020). However, the association between various types of ICSs and the pneumonia risk remains controversial, as the conclusions of the previous published meta-analyses are different (Sin et al., 2009; Singh et al., 2009; Kew and Seniukovich, 2014; Festic et al., 2016; Yang et al., 2019). However, the reliability and generalizability of these studies might be weakened by their small sample size, since a large number of important randomized controlled trials (RCTs) after 2017 were not included in these meta-analyses (Bhatt et al., 2017; Papi et al., 2017; Siler et al., 2017; Vestbo et al., 2017; Betsuyaku et al., 2018; Chapman et al., 2018; Ferguson et al., 2018a, Ferguson et al., 2018b; Frith et al., 2018; Lipson et al., 2018; Papi et al., 2018; Ichinose et al., 2019; Kerwin et al., 2019; Rabe et al., 2020). Moreover, none of these studies assessed the difference in the pneumonia risk in COPD patients with different demographic characteristics (including severity of airflow limitation, age, body mass index [BMI], etc.).

The aim of this meta-analysis was to objectively reappraise the pneumonia risk and serious pneumonia associated with various types of ICSs in COPD patients through all available RCTs. We also aimed to assess the impact of medication details (including dosage level and treatment duration) and demographic characteristics (severity, age, and body mass index) of patients on this association.

## Methods

### Protocol and Guidance

This meta-analysis was carried out according to the Preferred Reporting Items for Systematic review and Meta-Analysis (Moher et al., 2009). Ethics committee approval is not applicable for this meta-analysis. The study was registered with PROSPERO prospectively (#CRD42020213586).

### Search Strategy

Two reviewers (Hong Chen and Jian Sun) independently searched the databases of PubMed, Embase, Cochrane Library, and Clinical Trials.gov from inception until February 2021, using the following terms: (“chronic obstructive pulmonary disease” OR “COPD” OR “pulmonary disease, chronic obstructive” or “chronic obstructive airway disease” OR “airflow obstruction, chronic” OR “chronic airflow obstruction” OR “chronic obstructive lung disease” OR “emphysema” OR “Bronchitis”) AND (“inhaled corticosteroids” OR “ICS” OR “budesonide” OR “fluticasone” OR “mometasone” OR “beclomethasone” OR “triamcinolone” OR “ciclesonide”). Articles in English were included. Disagreements regarding eligibility were resolved by discussion by two investigators and, if necessary, consultation with a third investigator (Hao Yan).

### Eligibility Criteria

We included eligible studies based on the PICOS (Participants [P], Interventions [I], Comparators [C], Outcomes [O], and Study design [S]) criteria (Shamseer et al., 2015): *1*) Participants: patients aged 40 yr or over, with stable, moderate (GOLD stage II) to very severe (GOLD stage IV) COPD. Patients with other respiratory diseases, such as asthma, bronchiectasis were excluded. *2*) Interventions: various types and doses of ICSs as the intervention treatment. *3*) Comparisons: non-ICSs treatment as a control treatment. *4*) Outcome: Pneumonia for this meta-analysis was defined as an adverse event based on the Medical Dictionary for Regulatory Activities (MedDRA, version 10.0) pneumonia-related preferred terms, including “pneumonia,” “lobar pneumonia,” “bronchopneumonia,” “pneumonia pneumococcal,” or “pneumonia staphylococcal” (Rennard et al., 2009; Sin et al., 2009). One or more of the above MedDRA terms reported in the adverse events list or the safety profiles by the RCTs would be identified as pneumonia data, and included in our analysis. Serious pneumonia was defined as a pneumonia leading to mechanical ventilation or death, or requiring hospital admission (Aaron et al., 2007; Pascoe et al., 2015). *5*) Study design: only RCTs were included. Non-RCTs, such as retrospective studies, reviews, case reports and case-control studie, were excluded.

### Data Extraction and Quality Assessment

Two reviewers (Qiang Huang and Yongqi Liu) independently identified references and extracted data from eligible RCTs. Any disagreements would be resolved by discussion to reach a consensus, and consulted a third reviewer if necessary. The risk of bias of the included RCTs was assessed by two independent reviewers (Hong Chen and Mengxin Yuan) using the Cochrane risk of bias tool (Higgins et al., 2011). Any disagreements would be resolved by discussion and consultation (Hao Yan).

### Subgroup Analyses

Subgroup analyses were conducted based on: *1*) types of ICSs (fluticasone, budesonide, mometasone furoate and beclomethasone); *2*) doses of ICSs (low-dose [defined as 100–250 μg/d of fluticasone propionate or equivalent], medium-dose [defined as >250–500 μg/d of fluticasone propionate or equivalent], and high-dose [defined as >500 μg/d of fluticasone propionate or equivalent]); *4*) treatment durations (short-term ICSs treatment [defined as ≤6 mo] and long-term ICSs treatment [defined as >6 mo]); *5*) severity (moderate COPD [GOLD stage II], severe COPD [GOLD stage III] and very severe COPD [GOLD stage IV]); *6*) age of patients (<65 yr old and ≥65 yr old); *7*) body mass index (BMI) of patients (≥25 and <25).

### Statistical Analysis

The Review Manager 5.3 software was used to calculate the pooled results. Considering Peto odds ratio (Peto OR) could provide the best confidence interval (CI) when events are rare (Bradburn et al., 2007), the pooled results for the comparison of ICSs treatment vs non-ICSs treatment were calculated using Peto ORs. Sensitivity analysis was performed after excluding those studies with high risk of bias. Subgroup analyes based on the baseline demographic characteristics (severity, age, and body mass index) of the patients were conducted using the individual patient level data, which was extracted from the baseline data of the included RCTs (mean or median for lung function, age and BMI). This method of analyzing the individual patient level data was used by Sobieraj et al. (Sobieraj et al., 2018) previously. A two tailed *p*-value <0.05 was considered to be statistically significant. Statistical heterogeneity was further measured using the I^2^ test, and I^2^ ≥50% indicated a substantial heterogeneity (Higgins et al., 2003).

## Results

### Eligible Trials and Study Descriptions

**FIGURE 1:**
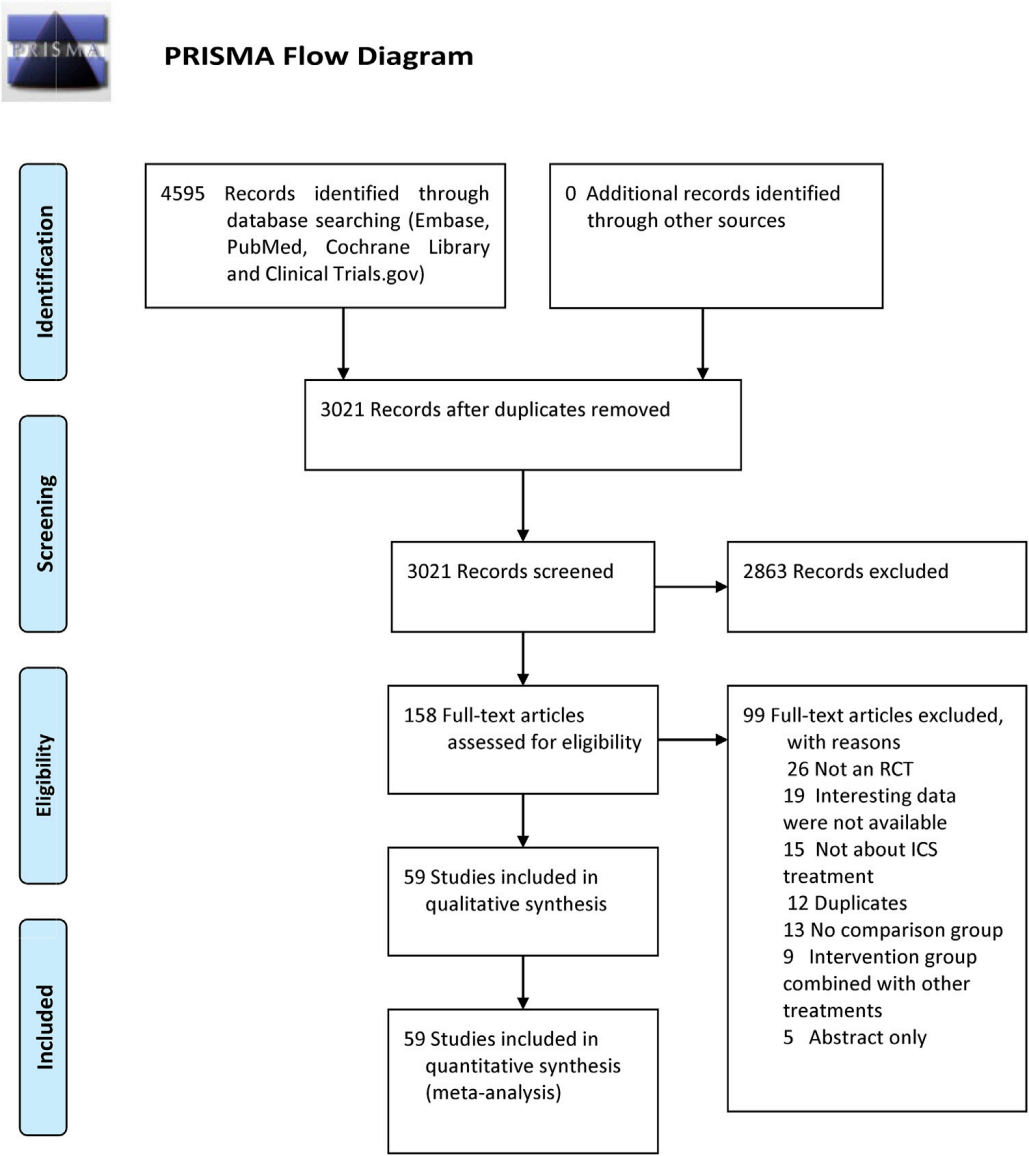
Literature search and screening process.

Our search identified 4,595 citations. After evaluating these citations, we included 59 RCTs (Vestbo et al., 1999; Burge et al., 2000; Calverley et al., 2003; Szafranski et al., 2003; Aaron et al., 2007; Calverley et al., 2007; Kardos et al., 2007; Calverley et al., 2008; Ferguson et al., 2008; Tashkin et al., 2008; Wedzicha et al., 2008; Anzueto et al., 2009; Rennard et al., 2009; Welte et al., 2009; Calverley et al., 2010; Dransfield et al., 2011; Doherty et al., 2012; Hanania et al., 2012; Jung et al., 2012; Sharafkhaneh et al., 2012; Tashkin et al., 2012a, Tashkin et al., 2012b; Dransfield et al., 2013; Fukuchi et al., 2013; Kerwin et al., 2013; Martinez et al., 2013; Vogelmeier et al., 2013; Magnussen et al., 2014; Ohar et al., 2014; Pepin et al., 2014; Rossi et al., 2014; Wedzicha et al., 2014; Donohue et al., 2015; Singh et al., 2015; Zheng et al., 2015; Zhong et al., 2015; Beeh et al., 2016; Covelli et al., 2016; Lee et al., 2016; Vestbo et al., 2016a; Vestbo et al., 2016b; Vogelmeier et al., 2016; Wedzicha et al., 2016; Bhatt et al., 2017; Ferguson et al., 2017; Papi et al., 2017; Siler et al., 2017; Vestbo et al., 2017; Betsuyaku et al., 2018; Chapman et al., 2018; Ferguson et al., 2018a; Ferguson et al., 2018b; Frith et al., 2018; Lipson et al., 2018; Papi et al., 2018; Huang et al., 2019; Ichinose et al., 2019; Kerwin et al., 2019; Rabe et al., 2020). These trials enrolled 103,477 patients, of whom 60,733 received ICSs treatment and 42,744 received non-ICSs treatment. The flowchart is shown in Figure 1. The studies included were published between 1999 and 2020, with sample size raging from 249 to 16,568 patients. All studies provided data on pneumonia, 14 of which provided data on serious pneumonia. Among the studies, 35 RCTs (67,109 patients) compared fluticasone and control, 17 RCTs (25,071 patients) compared budesonide and control, four RCTs (5,413 patients) compared mometasone and control, and four RCTs (5,884 patients) compared beclomethasone and control, respectively. No RCTs investigated triamcinolone or ciclesonide and the pneumonia risk in COPD patients. The detailed characteristics of the included RCTs are summarized in Table 1.

**TABLE 1:** Detailed characteristics of the included randomized controlled trials.

Study	Mean Age, y	Male, %	Postbron-chodilator FEV_1_ (% predicted)	Tobacco use, Pack-years	BMI	Duration, months	Interventions, μg	Pneumonia incidence	Serious pneumonia incidence
								Events/Patients	Events/Patients
Vestbo et al. (1999)	ICS: 59	ICS: 58.6	ICS: 86·2	NR	NR	36	ICS: BUD 400 bid	ICS: 16/145	NR
	CP: 59.1	CP: 62.1	CP: 86·9				CP: P	CP: 24/145	
Burge et al. (2000)	ICS: 63.8	ICS: 75.4	ICS: 50	44	ICS: 24.5	36	ICS: FP 500 bid	ICS: 20/376	ICS: 16/370
	CP: 63.7	CP: 75	CP: 50.3		CP: 24.9		CP: P	CP: 9/375	CP: 8/368
Calverley et al. (2003)	ICS: ≥40	ICS: 76	36	ICS: 39	NR	12	ICS: BUD/FM 320/9 bid; BUD 400 bid	ICS: 13/511	NR
	CP: ≥40	CP: 75		CP: 38.5			CP: FM 9 bid; P	CP: 9/511	
Szafranski et al. (2003)	ICS: 64	ICS: 78	ICS: 36.5	ICS: 44	NR	12	ICS: BUD 160/4.5 bid	ICS: 6/208	NR
	CP: 65	CP: 79.5	CP: 36	CP: 45			CP: FM 4.5 bid; P	CP: 9/205	
Aaron et al. (2007)	ICS: 67.8	ICS: 57.9	ICS: 42.2	ICS: 50.3	ICS: 27.8	12	ICS: SFC 250/25 bid	ICS: 1/145	ICS: 1/145
	CP: 67.9	CP: 56.6	CP: 41.6	CP: 50.3	CP: 27.4		CP: S	CP: 1/304	CP: 1/304
Calverley et al. (2007)	ICS: 65	ICS: 75	ICS: 44.2	ICS: 48.1	ICS: 25.4	36	ICS: SFC 500/50 bid; FP 500 bid	ICS: 217/3098	NR
	CP: 65.1	CP: 76	CP: 43.9	CP: 49	CP: 25.4		CP: S 50 bid; P	CP: 124/3086	
Kardos et al. (2007)	ICS: 63.8	ICS: 74	ICS: 40.4	ICS: 36.8	NR	10	ICS: SFC 500/50 bid	ICS: 23/507	NR
	CP: 64	CP: 77.6	CP: 40.3	CP: 37			CP: S 50 bid	CP: 7/487	
Calverley et al. (2008)	ICS: 65	ICS: 68	ICS: 46.5	NR	ICS: 26.4	12	ICS: MF 800 qd	ICS: 25/616	NR
	CP: 65	CP: 69	CP: 47		CP: 27.1		CP: P	CP: 6/295	
Ferguson et al. (2008)	ICS: 64.9	ICS: 58	ICS: 39.8	ICS: 58.5	ICS: 27.3	12	ICS: SFC 250/25 bid	ICS: 29/394	ICS: 19/394
	CP: 65.0	CP: 52	CP: 40.6	CP: 54.4	CP: 27.7		CP: S 25 bid	CP:15/388	CP: 10/388
Tashkin et al. (2008)	ICS: 63.3	ICS: 68.6	ICS: 39.4	ICS: 40.8	NR	6	ICS: BUD/FM 320/9 bid; BUD 160/9 bid	ICS: 8/1120	ICS: 8/1120
	CP: 63.4	CP: 67.3	CP: 40.4	CP: 40			CP: FM 9 bid; P	CP: 2/584	CP: 2/584
Wedzicha et al. (2008)	ICS: 64	ICS: 81	ICS: 39.1	ICS: 41.3	ICS: 20–29	24	ICS: SFC 500/50 bid	ICS: 50/658	ICS: 41/658
	CP: 65	CP: 84	CP: 39.4	CP: 39.5	CP: 20–29		CP: Tio 18 qd	CP: 24/665	CP: 19/665
Anzueto et al. (2009)	ICS: 65.4	ICS: 51	ICS: 41.2	ICS: 57.8	ICS: 27.6	12	ICS: SFC 250/25 bid	ICS: 26/394	NR
	CP: 65.3	CP: 57	CP: 40	CP: 56.5	CP: 27.3		CP: S 25 bid	CP: 10/403	
Rennard et al. (2009)	ICS: 63.4	ICS: 62.6	ICS: 39.1	40	NR	12	ICS: BUD/FM 320/9 bid; BUD/FM 160/9 bid	ICS:30/988	NR
	CP: 62.9	CP: 65.3	CP: 40				CP: FM 9 bid; P	CP: 40/976	
Welte et al. (2009)	ICS: 62.5	ICS: 76	ICS: 38.1	ICS: 36	ICS: 26.4	3	ICS: BUD/FM 320/9 bid + Tio 18 qd	ICS: 3/331	NR
	CP: 62.4	CP: 74	CP: 37.7	CP: 38	CP: 26.3		CP: Tio 18 qd + P	CP: 3/329	
Calverley et al. (2010)	ICS: 63.5	ICS: 80.4	NR	ICS: 37.6 CP: 39.7	NR	11	ICS: BDP/FM 200/24 bid; BUD/FM 400/24 bid	ICS: 5/232; 7/238	NR
	CP: 63.7	CP: 81.1					CP: FM	CP: 1/233	
Dransfield et al. (2011)	ICS: 63.6	ICS: 55	ICS: 56	ICS: 55.8	ICS: 26.7	3	ICS: SFC 250/50 bid	ICS: 3/123	NR
	CP: 63.5	CP: 59	CP: 55	CP: 54.1	CP: 26.6		CP: P	CP: 0/126	
Doherty et al. (2012)	ICS: 60.3	ICS: 75.3	ICS: 39	ICS: 45.4	NR	12	ICS: MF/FM 400/10 bid; MF/F 200/10 bid; MF 400 bid	ICS:16/717	NR
	CP: 59.2	CP: 75	CP: 38.1	CP: 44.7			CP: FM 10 bid; P	CP: 6/479	
Hanania et al. (2012)	ICS: 61.3	ICS: 50	ICS: 56	ICS: 55.4	ICS: 27	6	ICS: SFC250/50 bid + Tio 18 qd	ICS: 2/173	NR
	CP: 61	CP: 43	CP: 57.4	CP: 54.7	CP: 27.6		CP: Tio 18 qd	CP: 0/169	
Jung et al. (2012)	ICS: 67	ICS: 97.3	ICS: 47.4	NR	ICS: 22.2	6	ICS: SFC250/50 bid + Tio 18 qd	ICS: 2/223	NR
	CP: 67.8	CP: 98.7	CP: 47.5		CP: 21.8		CP: Tio 18 qd	CP: 2/232	
Sharafkhaneh et al., 2012	ICS: ≥40	ICS: 64.6	ICS: 37.8	ICS: 45	NR	12	ICS: BUD/FM 320/9 bid; BUD/FM 160/9 bid	ICS: 45/815	NR
	CP: ≥40	CP: 56.8	CP: 37.5	CP: 43			CP: FM	CP: 11/403	
Tashkin et al. (2012a)	ICS: 60.2	ICS: 76.7	ICS: 39.2	ICS: 43	NR	12	ICS: MF/F 400/10 bid; MF/F 200/10 bid; MF 400 bid	ICS: 19/1351	NR
	CP: 59.3	CP: 77	CP: 38.8	CP: 42.4			CP: FM 10 bid; P	CP: 9/900	
Tashkin et al. (2012b)	ICS: 60.1	ICS: 78.3	≥25 and ≤60	ICS: 40.5	NR	12	ICS: MF/F 400/10 bid; MF/F 200/10 bid; MF 400 bid	ICS: 3/634	NR
	CP: 59.7	CP: 76.5		CP: 40.3			CP: FM 10 bid; P	CP: 3/421	
Dransfield et al. (2013)	ICS: 63.6	ICS: 57	ICS: 45.6	NR	NR	12	ICS: FF/VI 200/25 qd; FF/VI 100/25 qd; FF/VI 50/25 qd	ICS: 154/2437	ICS: 71/2378
	CP: 63.8	CP: 57	CP: 45.2				CP: VI 25 qd	CP: 27/818	CP: 8/799
Fukuchi et al. (2013)	ICS: 64.5	ICS: 87.6	ICS: 40.9	ICS: 44.4	NR	3	ICS: BUD/FM 160/4.5 bid	ICS: 8/636	NR
	CP: 65.6	CP: 90.3	CP: 40.8	CP: 44.7			CP: FM 4.5 bid	CP: 7/657	
Kerwin et al. (2013)	ICS: 62.6	ICS: 65.7	ICS: 47.7	ICS: 45.7	NR	6	ICS: FF/VI 100/25qd; FF/VI 50/25 qd	ICS: 12/618	NR
	CP: 62.8	CP: 68	CP: 49.2	CP: 46.6			CP: VI 25 qd; P	CP: 8/412	
Martinez et al. (2013)	ICS: 61.7	ICS: 71.5	ICS: 47.7	ICS: 41.9	NR	6	ICS: FF/VI 200/25 qd; FF/VI 100/25 qd	ICS: 10/816	NR
	CP: 61.7	CP: 74	CP: 48.4	CP: 43.9			CP: VI 25 qd; P	CP: 2/408	
Vogelmeier et al. (2013)	ICS: 63.2	ICS: 71.6	ICS: 60.5	NR	NR	6	ICS: SFC 500/50 bid	ICS: 4/264	ICS: 2/264
	CP: 63.4	CP: 70.2	CP: 60				CP: IND/GLY 110/50 qd	CP: 0/258	CP: 0/258
Magnussen et al. (2014)	ICS: 63.6	ICS: 81.5	<50	NR	NR	12	ICS: SFC 500/50 bid + Tio 18 qd	ICS: 72/1243	NR
	CP: 64	CP: 83.4					CP: S 50 bid + Tio 18 qd	CP: 68/1242	
Ohar et al. (2014)	ICS: 63.1	ICS: 55	ICS: 38.5	ICS: 52	ICS: 28	6	ICS: SFC 250/50 bid	ICS: 13/314	NR
	CP: 62.7	CP: 54	CP: 41.2	CP: 55.3	CP: 28.3		CP: S 50 bid	CP: 10/325	
Pepin et al. (2014)	ICS: 66.7	ICS: 85	ICS: 45.6	ICS: 42.6	ICS: 27.1	3	ICS: FF/VI 100/25 qd	ICS: 3/127	NR
	CP: 67.7	CP: 86	CP: 47.4	CP: 44.6	CP: 27.2		CP: Tio 18 qd	CP: 0/130	
Rossi et al. (2014)	ICS: 66.8	ICS: 68.4	ICS: 62.4	ICS: 42	NR	6	ICS: SFC 500/50 bid	ICS: 2/288	NR
	CP: 65.3	CP: 69.6	CP: 64	CP: 41.4			CP: IND/GLY 110/50 qd	CP: 0/293	
Wedzicha et al. (2014)	ICS: 64.6	ICS: 69	ICS: 41.9	ICS: 43.1	ICS: 26.5	11	ICS: BDP/FM 100/6 bid	ICS: 23/601	NR
	CP: 63.9	CP: 69	CP: 41.6	CP: 42.7	CP: 26.5		CP: FM 6 bid	CP: 11/596	
Donohue et al. (2015)	ICS: 63.0	ICS: 72.5	ICS: 49.6	ICS: 38.3	ICS: 27	3	ICS: SFC 250/50 bid	ICS: 8/701	NR
	CP: 62.5	CP: 74	CP: 49.3	CP: 37.8	CP: 27.5		CP: UMEC/VI 62.5/25 qd	CP: 3/702	
Singh et al. (2015)	ICS: 61.4	ICS: 71	ICS: 51.1	ICS: 37.7	NR	3	ICS: SFC 500/50 bid	ICS: 0/358	NR
	CP: 61.8	CP: 73	CP: 50.2	CP: 37.8			CP: UMEC/VI 62.5/25 qd	CP: 1/358	
Zheng et al. (2015)	ICS: 64.4	ICS: 91.3	ICS: 48.4	ICS: 38	NR	5.6	ICS: FF/VI 200/25 qd; FF/VI 100/25 qd; FF/VI 50/25 qd	ICS: 8/480	BMI <25
	CP: 64.7	CP: 90	CP: 48.6	CP: 43.3			CP: P	CP: 4/162	
Zhong et al. (2015)	ICS: 65.3	ICS: 89.7	ICS: 52	NR	NR	6	ICS: SFC 500/50 bid	ICS: 10/369	NR
	CP: 64.8	CP: 91.7	CP: 51.6				CP: IND/GLY 110/50 qd	CP: 3/372	
Beeh et al. (2016)	63.6	64.6	56.4	39.1	NR	1.5	ICS: SFC 50/500 bid; SFC 50/250 bid	ICS: 2/431	NR
							CP: Tio/Olo 5/5 qd; Tio/Olo 2.5/5 qd	CP: 3/436	
Covelli et al. (2016)	ICS: 62.9	ICS: 62	≥30 and ≤70	ICS: 43.2	ICS: 28.4	3	ICS: FF/VI 100/25 qd	ICS: 3/310	NR
	CP: 62.3	CP: 67		CP: 45.6	CP: 28.6		CP: Tio 18 qd	CP: 0/313	
Lee et al. (2016)	ICS: 66.8	ICS: 97.2	ICS: 35.8	NR	ICS: 21.3	3	ICS: BUD/FM 320/9 bid + Tio 18 qd	ICS: 2/289	NR
	CP: 66.9	CP: 94.1	CP: 37		CP: 21.2		CP: Tio 18 qd	CP: 4/289	
Vestbo et al. (2016a)	ICS: 65.0	ICS: 25	ICS: 59.7	ICS: 41	ICS: 28	22	ICS: FF/VI 100/25 qd	ICS: 465/8297	NR
	CP: 65.1	CP: 25.5	CP: 59.7	CP: 41	CP: 28		CP: VI 25 qd; P	CP: 377/8271	
Vestbo et al. (2016b)	ICS: 67	ICS: 50	NR	NR	ICS: 28	12	ICS: FF/VI 100/25 qd	ICS: 94/1396	NR
	CP: 67	CP: 52			CP: 28		CP: P	CP: 83/1403	
Vogelmeier et al. (2016)	ICS: 63.5	ICS: 64.4	ICS: 53.2	ICS: 42.6	NR	6	ICS: SFC 50/500 bid	ICS: 9/466	NR
	CP: 63.3	CP: 65.7	CP: 53.3	CP: 41.6			CP: ACL/FM 400/12 bid	CP: 3/467	
Wedzicha et al. (2016)	ICS: 64.5	ICS: 74.8	ICS: 44.1	NR	NR	12	ICS: SFC 50/500 bid	ICS: 80/1680	NR
	CP: 64.6	CP: 77.3	CP: 44				CP: IND/GLY 110/50 qd	CP: 53/1678	
Bhatt et al. (2017)	ICS: 68.5	ICS: 77	≤70	ICS: 50.1	ICS: 24.5	6	ICS: FF/VI 100/25 qd	ICS: 2/141	NR
	CP: 68.5	CP: 80.5		CP: 49.4	CP: 24.6		CP: VI 25 qd; P	CP: 3/303	
Ferguson et al. (2017)	ICS: 63.1	ICS: 58.6	ICS: 48.5	ICS: 39	NR	6	ICS: BUD/FM 320/9 bid	ICS: 3/605	ICS: 0/605
	CP: 63.9	CP: 56	CP: 48.9	CP: 40			CP: FM 9 bid	CP: 6/613	CP: 5/613
Papi et al. (2017)	ICS: 63.4	ICS: 72.6	ICS: 37.9	ICS: 47.4	NR	12	ICS: FP/FM 500/20; FP/FM 250/10	ICS: 40/1175	NR
	CP: 64	CP: 75.9	CP: 37.7	CP: 50			CP: FM 12 bid	CP: 11/590	
Siler et al. (2017)	ICS: 65.3	ICS: 75	ICS: 50.3	ICS: 43.7	NR	3	ICS: FF/VI 100/25 qd	ICS: 7/806	NR
	CP: 65.4	CP: 77	CP: 50.5	CP: 44.1			CP: VI 25 qd	CP: 7/814	
Vestbo et al. (2017)	ICS: 63	ICS: 75.5	ICS: 36.7	NR	ICS: 26.4	12	ICS: BDP/FM 100/12.5 bid + Tio 18 qd; BDP/FM 100/6 bid + Tio 18 qd	ICS:40/1614	ICS: 30/1614
	CP: 63.3	CP: 77	CP: 36.6		CP: 26.2		CP: Tio 18 qd	CP: 19/1076	CP: 14/1076
Betsuyaku et al. (2018)	ICS: 68.6	ICS: 94	ICS: 59.5	ICS: 60.8	NR	6	ICS: SFC 50/250 bid	ICS:6/204	ICS: 4/204
	CP: 68	CP: 96	CP: 57.8	CP: 54.5			CP: Tio 18 qd	CP: 6/201	CP: 2/201
Chapman et al. (2018)	ICS: 65.3	ICS: 69.4	ICS: 57	NR	ICS: 28.2	6	ICS: SFC 50/500 bid	ICS: 9/526	NR
	CP: 65.5	CP: 71.7	CP: 56.2		CP: 27.8		CP: IND/GLY 110/50 qd	CP: 6/527	
Ferguson et al. (2018a)	ICS: 64.3	ICS: 60.5	ICS: 52.9	ICS: 44.6	ICS: 28.3	6	ICS: BUD/FM 320/10 bid; BUD/FM 160/10 bid; BUD/FM 400/12 bid; BUD 320 bid	ICS: 16/1717	ICS: 12/1717
	CP: 64.1	CP: 59.5	CP: 52.6	CP: 44.9	CP: 28.4		CP: FM 10 bid	CP: 9/644	CP: 6/644
Ferguson et al. (2018b)	ICS: 65.3	ICS: 72.4	ICS: 50.3	ICS: 45	ICS: 26.2	6	ICS: BGF 320/18/9.6 bid; BFF 320/9.6 bid; BFF 400/12 bid	ICS: 22/1271	NR
	CP: 65.1	CP: 68.8	CP: 50.2	CP: 45	CP: 26.3		CP: GFF 18/9.6 bid	CP: 10/625	
Frith et al. (2018)	ICS: 65.1	ICS: 89.6	ICS: 51.7	ICS: 45.3	ICS: 24.6	3	ICS: SFC 50/500 bid	ICS: 1/250	NR
	CP: 65	CP: 88.7	CP: 51.3	CP: 44.3	CP: 24.5		CP: IND/GLY 110/50 qd	CP: 1/248	
Lipson et al. (2018)	ICS: 65.3	ICS: 66.6	ICS: 45.6	NR	ICS: 26.6	12	ICS: FF/UMEC/VI 100/62.5/25 qd; FF/VI 100/25 qd	ICS: 609/8285	ICS: 336/8285
	CP: 65.2	CP: 66	CP: 45.4		CP: 26.7		CP: UMEC/VI 62.5/25 qd	CP: 97/2070	CP: 54/2070
Papi et al. (2018)	ICS: 64.4	ICS: 72	ICS: <50	NR	ICS: 25.7	12	ICS: BDP/FM/GLY 87/5/9 bid	ICS: 28/764	ICS: 18/764
	CP: 64.5	CP: 72	CP: <50		CP: 26.6		CP: IND/GLY 85/43 qd	CP: 27/768	CP: 17/768
Huang et al. (2019)	ICS: 63.8	ICS: 87.6	NR	ICS: 33.4	NR	3	ICS: BUD/F 320/9 bid + I/T	ICS: 1/293	NR
	CP: 64.4	CP: 85.3		CP: 32.7			CP: I/T	CP: 0/289	
Ichinose et al. (2019)	ICS: 69.7	ICS: 95.7	ICS: 51.8	ICS: 51.8	ICS: 22.9	6	ICS: BGF 320/18/9.6 bid; BFF 320/9.6 bid; BFF 400/12 bid	ICS: 19/278	NR
	CP: 69	CP: 97.1	CP: 52.2	CP: 52	CP: 22.5		CP: GFF 18/9.6 bid	CP: 5/138	
Kerwin et al. (2019)	ICS: 63.3	ICS: 55	≥25 and <80	ICS: 45.8	ICS: 29	6	ICS: BGF 320/18/9.6 bid; BFF 320/9.6 bid	ICS: 2/282	ICS: 2/282
	CP: 62.4	CP: 50		CP: 50	CP: 29		CP: GFF 18/9.6 bid	CP: 4/174	CP: 4/174
Rabe et al. (2020)	ICS: 64.6	ICS: 60.1	ICS: 43.4	ICS: 47.3	NR	12	ICS: BGF 320/18/9.6 bid; BGF 160/18/9.6 bid; BFF 320/9.6 bid	ICS: 261/6404	NR
	CP: 64.8	CP: 58.7	CP: 43.5	CP: 48.4			CP: GFF 18/9.6 bid	CP: 48/2125	

RCT, randomized controlled trial; FEV_1_, forced expiratory volume in the first second; BMI, body-mass index; ICSs, inhaled corticosteroids; CP, control or placebo; BUD, budesonide; NR, not reported; bid, twice daily; qd, once daily; FP, fluticasone propionate; S, salmeterol; SFC, fluticasone propionate/salmeterol; EOC, eosinophil counts; BUD/FM, budesonide/formoterol fumarate; FM, formoterol fumarate; MF, mometasone furoate; Tio, tiotropium/olodaterol; BDP, Beclomethasone dipropionate; FF/VI, fluticasone furoate/vilanterol; VI, vilanterol; UMEC/VI, umeclidinium/vilanterol; ACL/FM, aclidinium/formoterol; IND/GLY, indacaterol/glycopyrronium; FF/UMEC/VI, fluticasone furoate/umeclidinium/vilanterol; BDP/FM/GLY, beclomethasone/formoterol/glycopyrronium; I/T, ipratropium/theophylline; BGF, budesonide/glycopyrronium/formoterol; BFF, budesonide/formoterol; GFF, glycopyrrolate/formoterol.

### Assessment of Risk of Bias

**FIGURE 2:**
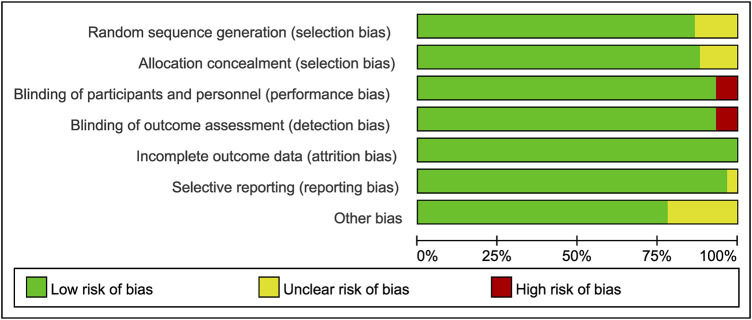
Risk of bias graph.

**FIGURE 3:**
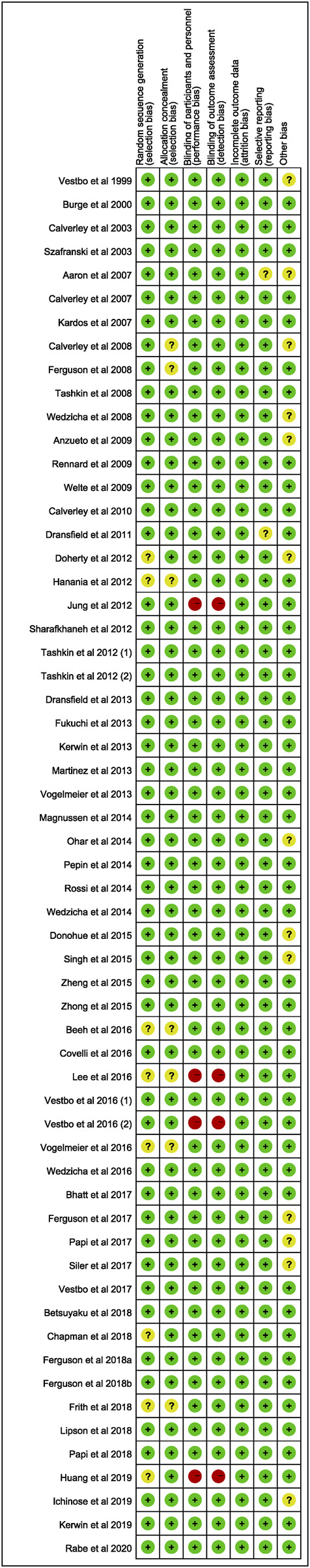
Risk of bias summary.

All included studies were assessed using the Cochrane Collaboration risk of bias assessment tool. The results are presented in Figures 2, 3. Thirty-five RCTs were assessed as being at low risk of bias for all aspects. Four had a high risk of bias for performance bias (blinding of participants and personnel) and detection bias (Blinding of outcome assessment). Twenty-two had an unclear risk for random sequence generation, selective reporting, allocation concealment, or other bias (Figures 2, 3).

### Various Types of ICSs and Pneumonia Risk

**FIGURE 4:**
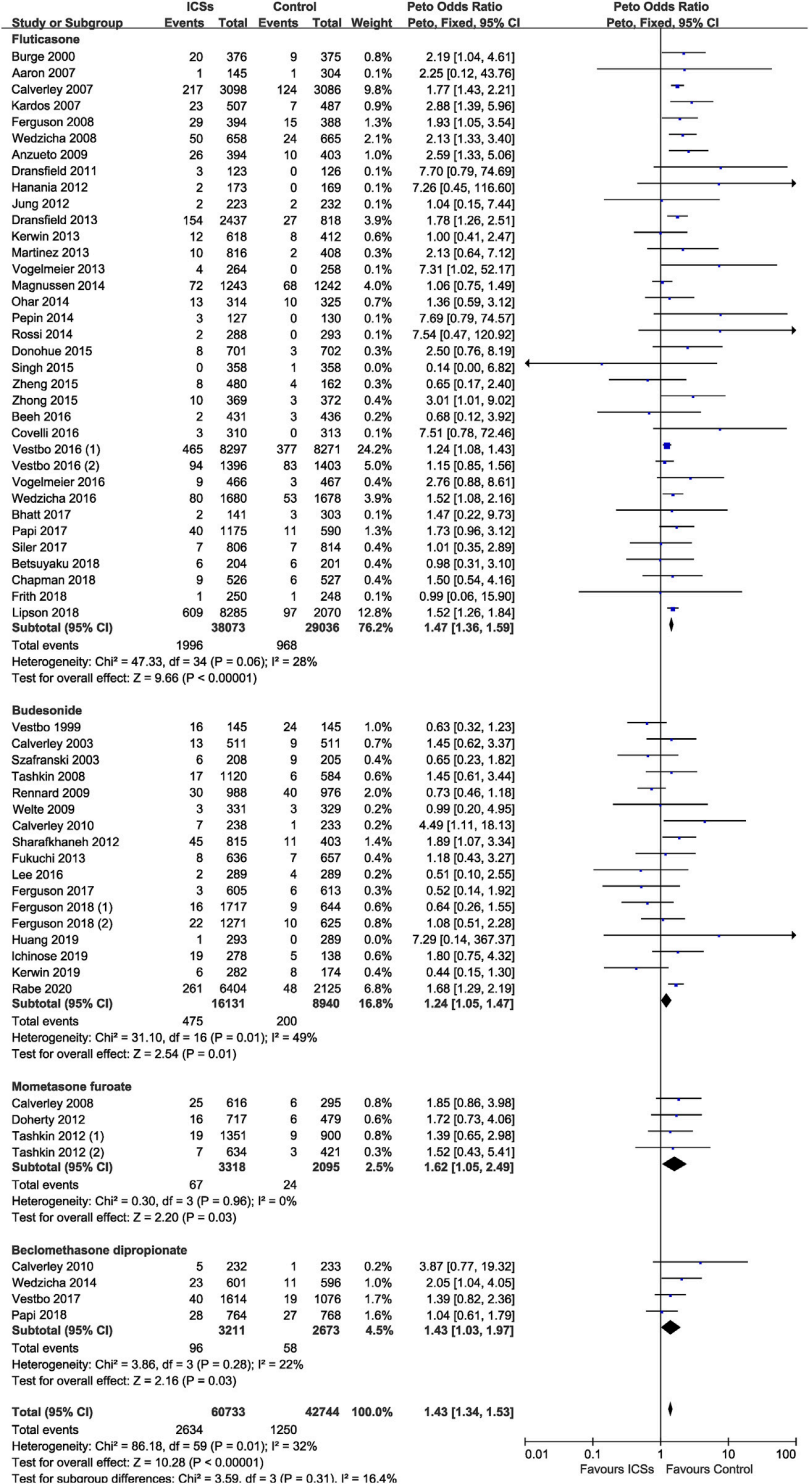
Various types of inhaled corticosteroids and pneumonia risk.

Compared with non-ICSs treatment, ICSs treatment significantly increased the pneumonia risk (Peto OR, 1.43; 95% CI, 1.34–1.53). Subgroup analysis based on types of ICSs showed that all types of ICSs increased the pneumonia risk ([fluticasone: Peto OR, 1.47; 95% CI, 1.36–1.59]; [budesonide: Peto OR, 1.24; 95% CI, 1.05–1.47]; [mometasone: Peto OR, 1.62; 95% CI, 1.05–2.49]; [beclomethasone: Peto OR, 1.43; 95% CI, 1.03–1.97]). Test for subgroup differences (I^2^ = 16.4%) indicated that there was no significant difference in the pneumonia risk associated with different types of ICSs (Table 2 and Figure 4).

**TABLE 2:** Summary of the pooled results.

Pooled results	No. of Patients	No. of Studies	Peto OR (95% CI)	Test for subgroup differences
Various types of ICSs and pneumonia risk				I^2^ = 16.4%
All types of ICSs	103,477	59	1.43 (1.34–1.53)	
Fluticasone	67,109	35	1.47 (1.36–1.59)	
Budesonide	25,071	17	1.24 (1.05–1.47)	
Mometasone furoate	5,413	4	1.62 (1.05–2.49)	
Beclomethasone dipropionate	5,884	4	1.43 (1.03–1.97)	
Different doses of ICSs and pneumonia risk				I^2^ = 74%
Low -dose	54,287	23	1.33 (1.22–1.45)	
Medium-dose	27,302	26	1.50 (1.28–1.76)	
High-dose	32,592	27	1.64 (1.45–1.85)	
Different treatment durations of ICSs and pneumonia risk				I^2^ = 0%
≤6 mo	26,408	31	1.30 (1.04–1.63)	
>6 mo	76,826	28	1.44 (1.34–1.55)	
ICSs associated pneumonia in COPD patients with different severity				I^2^ = 90.1%
Moderate COPD (GOLD stage II)	30,809	18	1.26 (1.11–1.43)	
Severe COPD (GOLD stage III)	65,773	34	1.54 (1.42–1.68)	
Very severe COPD (GOLD stage IV)	1148	2	2.52 (1.88–3.38)	
ICSs associated pneumonia in COPD patients with different age				I^2^ = 0%
<65 yr	50,802	35	1.43 (1.28–1.60)	
≥65 yr	46,963	19	1.41 (1.29–1.54)	
ICSs associated pneumonia in COPD patients with different BMI				I^2^ = 0%
<25	4,443	8	1.47 (1.02–2.12)	
≥25	54,867	23	1.43 (1.31–1.55)	
Various types of ICSs and serious pneumonia risk				I^2^ = 61.9%
All types of ICSs	29,008	15	1.55 (1.31–1.84)	
Fluticasone	17,091	7	1.75 (1.44–2.14)	
Budesonide	7,695	6	1.06 (0.68–1.65)	
Beclomethasone dipropionate	4,222	2	1.24 (0.79–1.95)	
Sensitivity analysis after excluding RCTs with high risk of bias (various types of ICSs and pneumonia risk)				I^2^ = 23.2%
All types of ICSs	99,063	55	1.45 (1.35–1.56)	
Fluticasone	63,855	33	1.50 (1.38–1.62)	
Budesonide	23,911	15	1.25 (1.06–1.48)	
Mometasone furoate	5,413	4	1.62 (1.05–2.49)	
Beclomethasone dipropionate	5,884	4	1.43 (1.03–1.97)	

ICSs, inhaled corticosteroids; Peto OR, Peto odds ratio; COPD, chronic obstructive pulmonary disease; GOLD, Global Initiative for Chronic Obstructive; BMI, body mass index.

### Different Doses of ICSs and Pneumonia Risk

**FIGURE 5:**
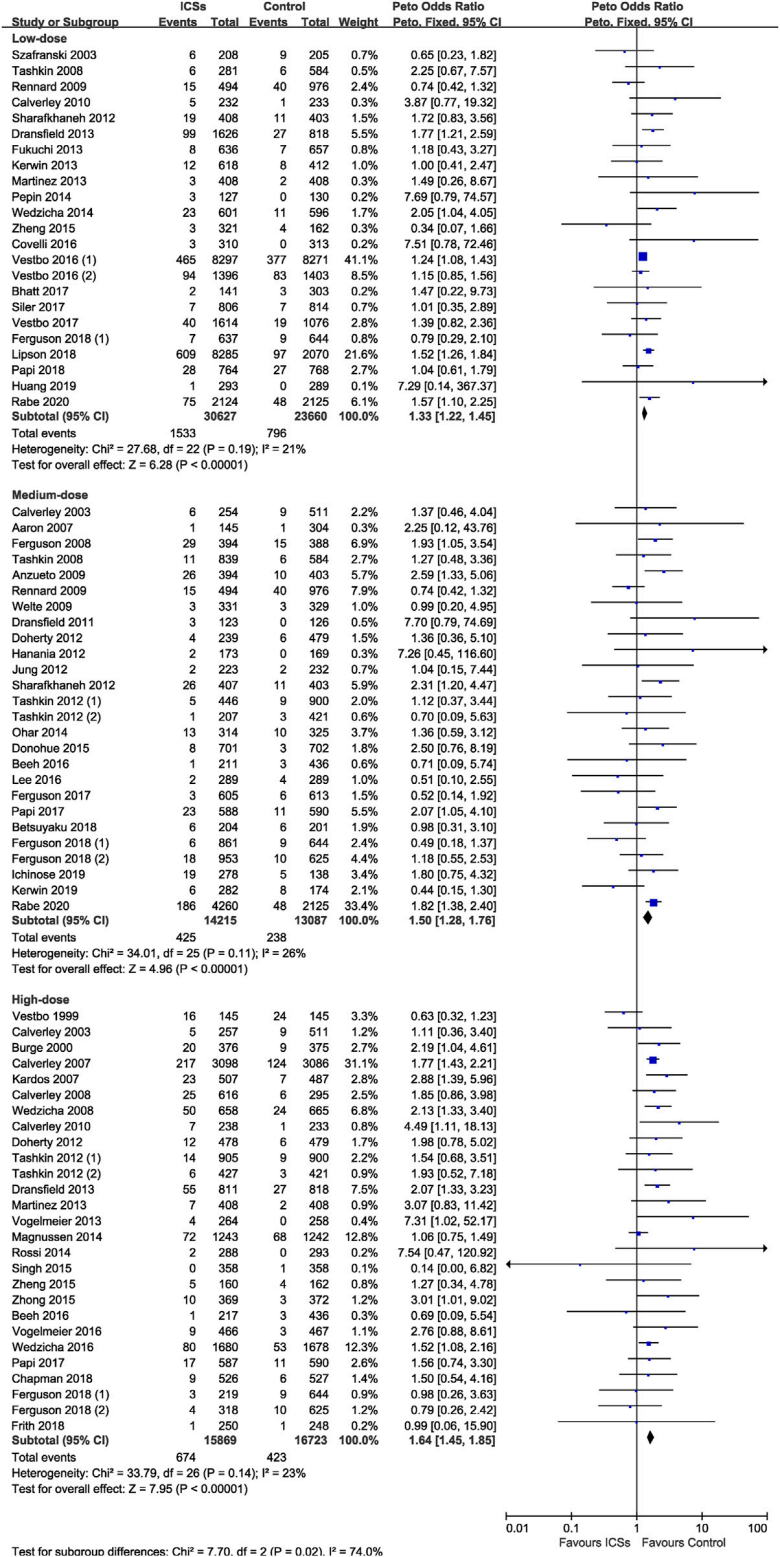
Different doses of inhaled corticosteroids and pneumonia risk.

Of the included trials, 23 RCTs (54,287 patients), 26 RCTs (27,302 patients), and 27 RCTs (32,592 patients) assessed high-dose, medium-dose, and low-dose ICSs and pneumonia risk, respectively. Subgroup analysis showed that there was a dose-response relationship between ICSs treatment and pneumonia risk. Low-dose (Peto OR, 1.33; 95% CI, 1.22–1.45), medium-dose (Peto OR, 1.50; 95% CI, 1.28–1.76), and high-dose (Peto OR, 1.64; 95% CI, 1.45–1.85) ICSs all significantly increased the pneumonia risk. Test for subgroup differences (I^2^ = 74%) indicated that there was a significant difference in the pneumonia risk associated with different doses of ICSs (Table 2 and Figure 5).

### Different Treatment Durations of ICSs and Pneumonia Risk

**FIGURE 6:**
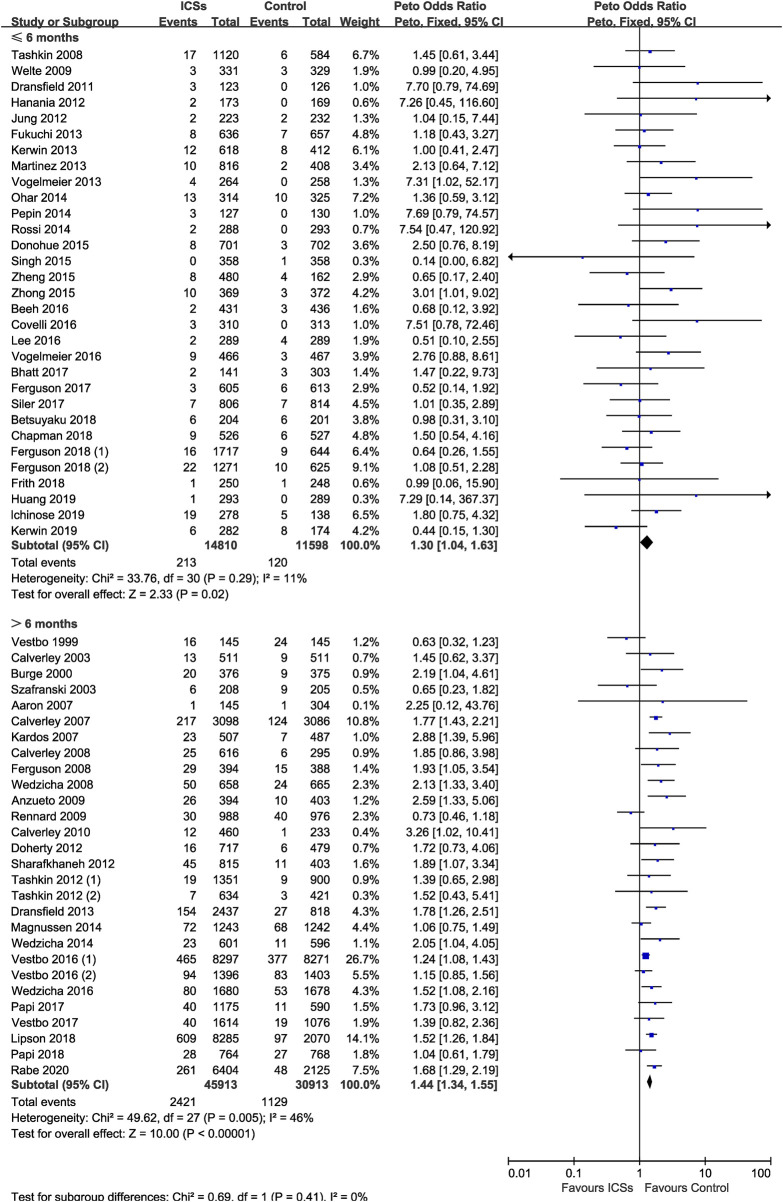
Different treatment durations of inhaled corticosteroids and pneumonia risk.

Of the included trials, 31 RCTs (26,408 patients), and 28 RCTs (76,826 patients) assessed short-term ICSs treatment and long-term ICSs treatment and pneumonia risk. Subgroup analysis showed that both short-term ICSs treatment (Peto OR, 1.30; 95% CI, 1.04–1.63) and long-term ICSs treatment (Peto OR, 1.44; 95% CI, 1.34–1.55) significantly increased the pneumonia risk. Test for subgroup differences (I^2^ = 0%) indicated that there was no significant difference in the pneumonia risk associated with different treatment durations of ICSs (Table 2 and Figure 6).

### ICSs Associated Pneumonia in COPD Patients With Different Severity

**FIGURE 7:**
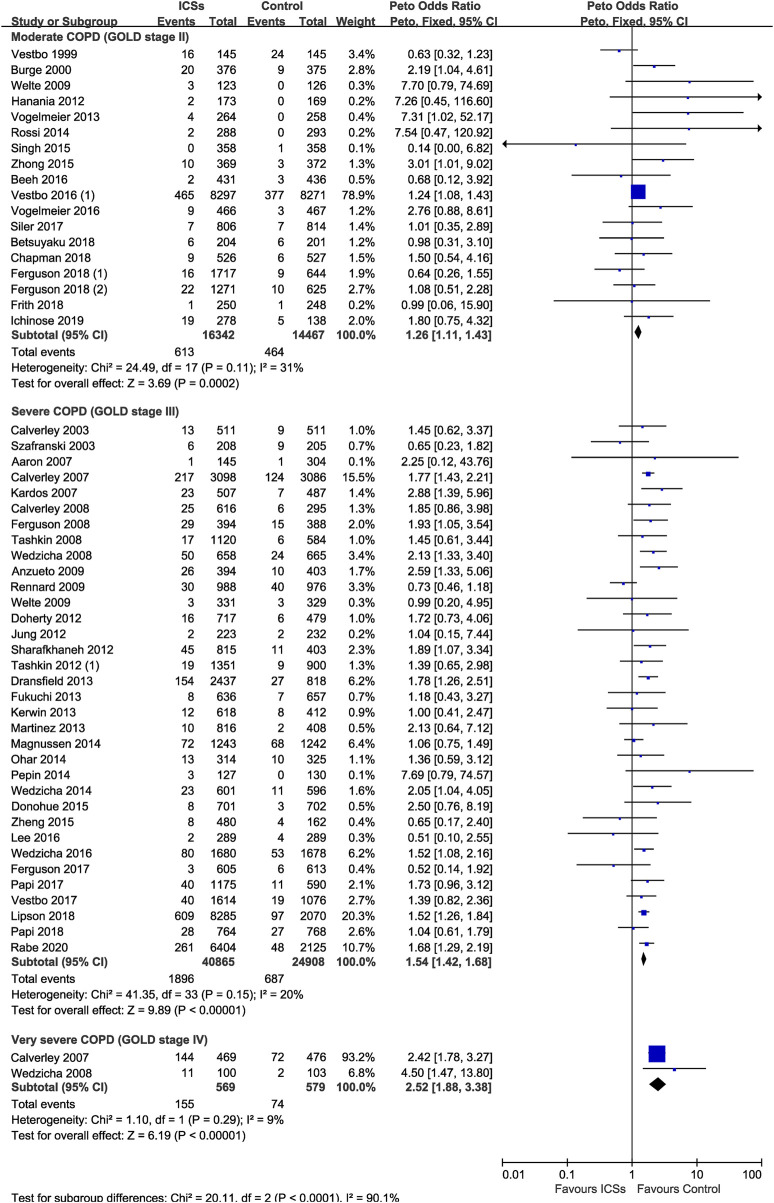
Inhaled corticosteroids associated pneumonia in COPD patients with different severity.

Eighteen RCTs (30,809 patients), 34 RCTs (65,773 patients) and two RCTs (1,148 patients) assessed ICSs associated pneumonia in moderate, severe, and very severe COPD patients, respectively. Subgroup analysis showed that the pneumonia risk was related with COPD severity. ICSs treatment significantly increased the pneumonia risk in all severity subgroups of COPD patients: ([Moderate COPD: Peto OR, 1.26; 95% CI, 1.11–1.43]; [Severe COPD: Peto OR, 1.54; 95% CI, 1.42–1.68]; [Very severe COPD: Peto OR, 2.52; 95% CI, 1.88–3.38]) (Table 2 and Figure 7). Test for subgroup differences (I^2^ = 90.1%) indicated that there was a significant difference in the pneumonia risk in patients with different severity.

### ICSs Associated Pneumonia in COPD Patients With Different Age

**FIGURE 8:**
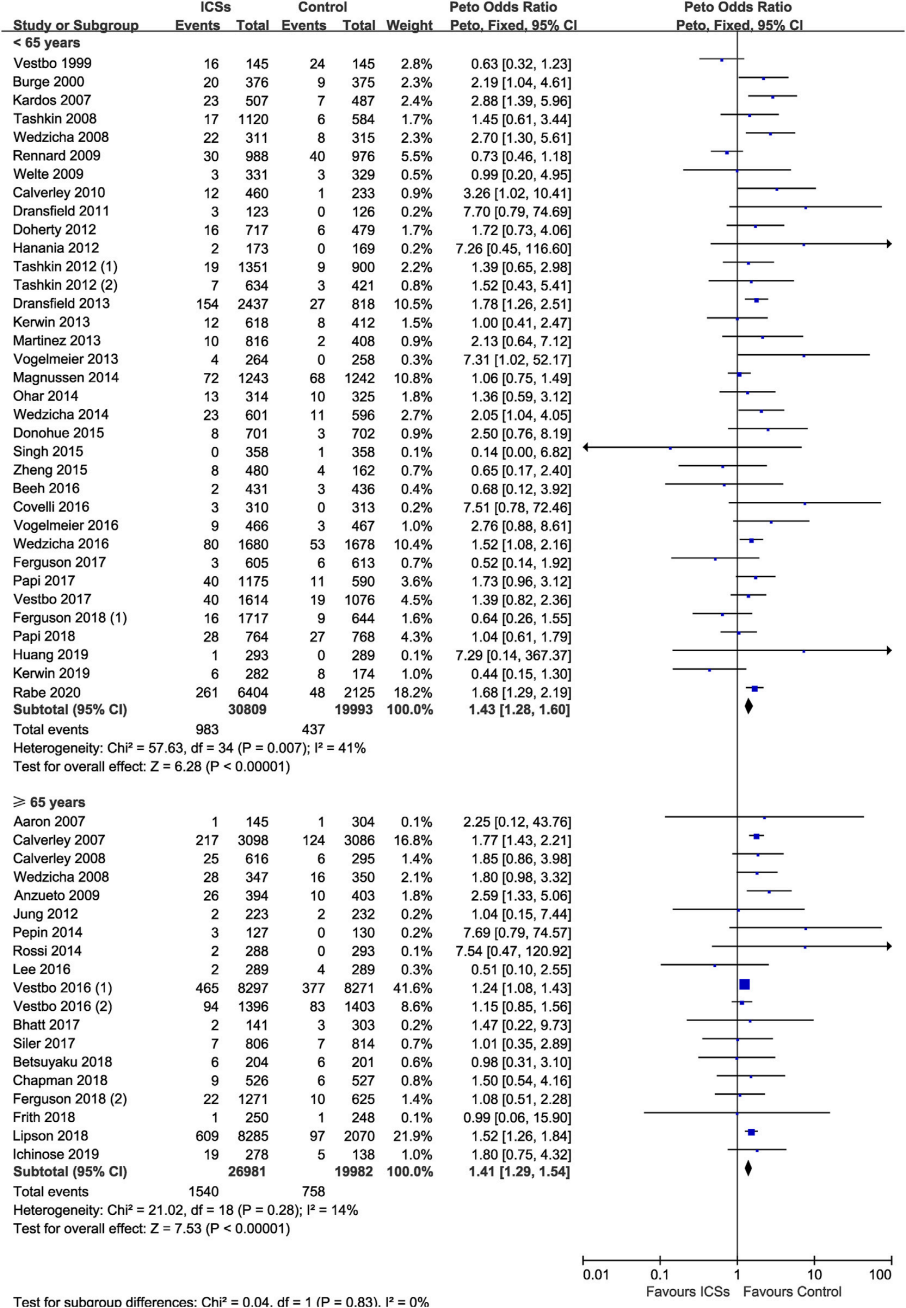
Inhaled corticosteroids associated pneumonia in COPD patients with different age.

Thirty-five RCTs (50,802 patients) and 19 RCTs (46,963 patients) assessed ICSs associated pneumonia in patients with different age. Subgroup analysis showed that ICSs treatment significantly increased the pneumonia risk in patients both age subgroups: ([<65 yr old: Peto OR, 1.43; 95% CI, 1.28–1.60]; [≥65 yr old: Peto OR, 1.41; 95% CI, 1.29–1.54]). Test for subgroup differences (I^2^ = 0%) indicated that there was no significant difference in the pneumonia risk in patients with different age (Table 2 and Figure 8).

### ICSs Associated Pneumonia in COPD Patients With Different BMI

**FIGURE 9:**
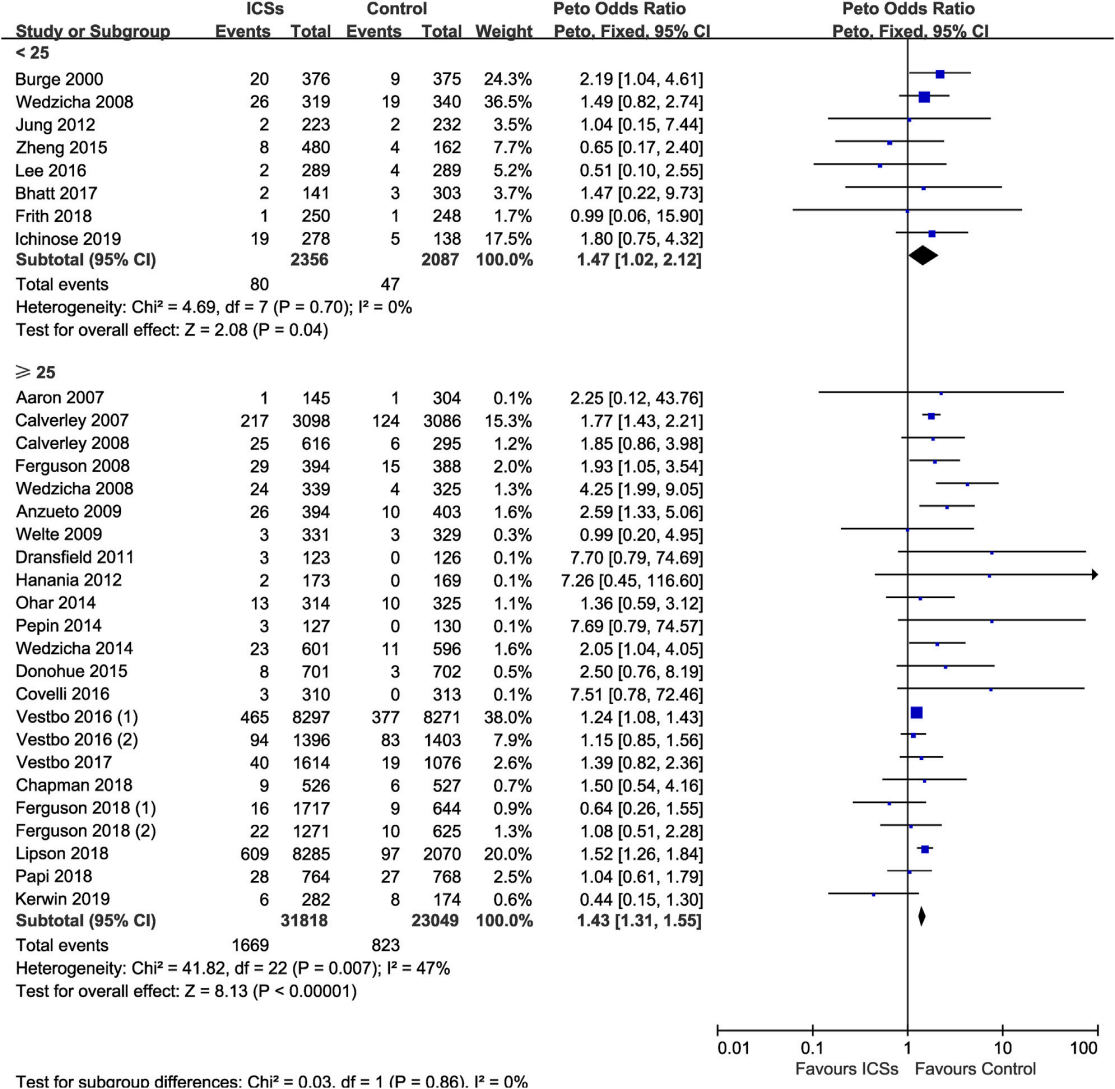
Inhaled corticosteroids associated pneumonia in COPD patients with different body mass index.

Eight RCTs (4,443 patients) and 23 RCTs (54,867 patients) assessed ICSs associated pneumonia in patients with different BMI. Subgroup analysis showed that ICSs treatment significantly increased the pneumonia risk in patients both BMI subgroups: ([<25: Peto OR, 1.47; 95% CI, 1.02–2.12]; [≥25: Peto OR, 1.43; 95% CI, 1.31–1.55]). Test for subgroup differences (I^2^ = 0%) indicated that there was no significant difference in the pneumonia risk in patients with different BMI (Table 2 and Figure 9).

### Various Types of ICSs and Serious Pneumonia Risk

**FIGURE 10:**
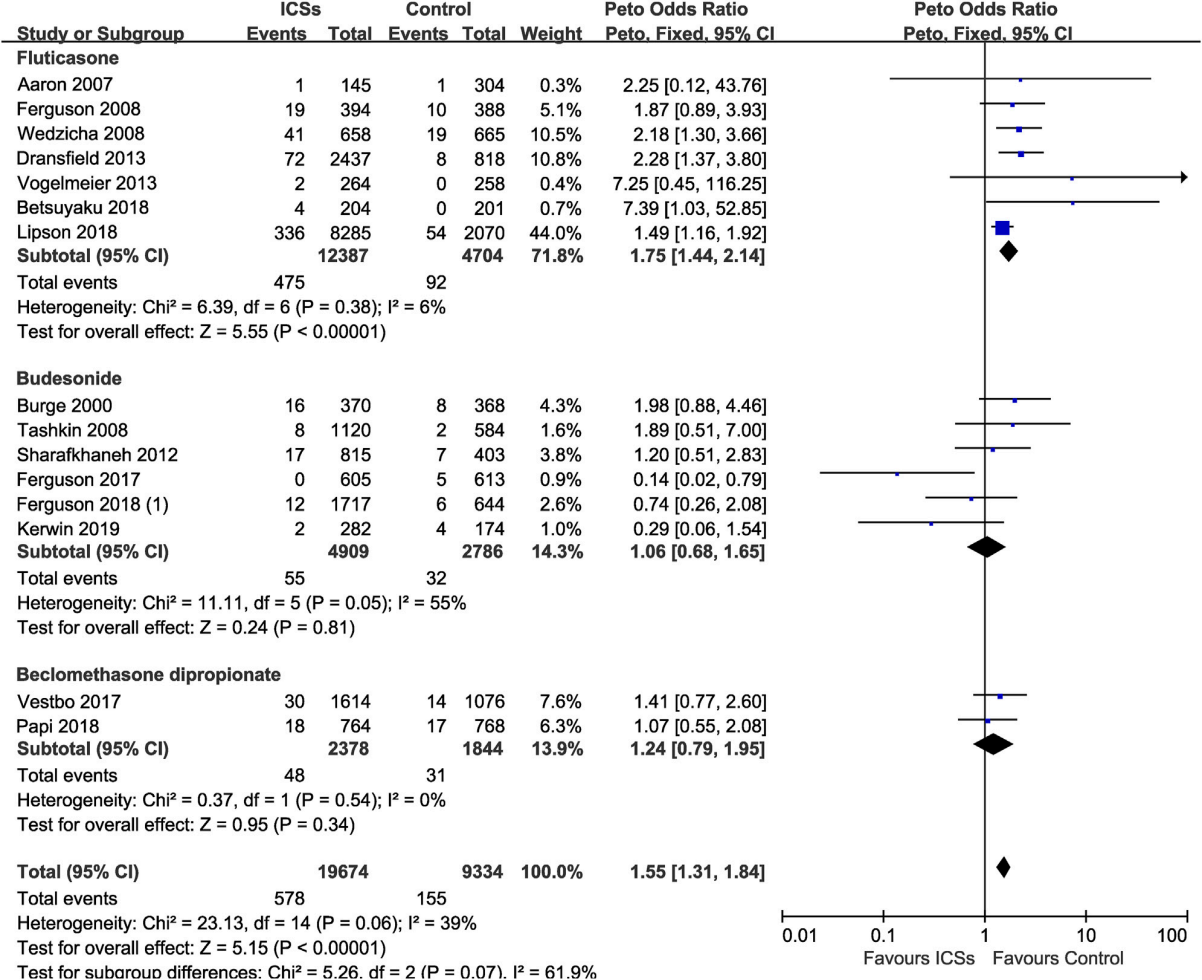
Various types of inhaled corticosteroids and serious pneumonia risk.

Of the included trials, 15 RCTs (29,008 patients) offered data on serious pneumonia associated with ICSs treatment. Compared with non-ICSs treatment, ICSs treatment significantly increased the serious pneumonia risk (Peto OR, 1.55; 95% CI, 1.31–1.84). Of the included RCTs, seven RCTs (17,091 patients) assessed fluticasone and serious pneumonia risk, six RCTs (7,695 patients) assessed budesonide and two RCTs (4,222 patients) assessed beclomethasone, respectively. Subgroup analysis showed that only fluticasone significantly increased the serious pneumonia risk (Peto OR, 1.75; 95% CI, 1.44–2.14) while budesonide (Peto OR, 1.06; 95% CI, 0.68–1.65) and beclomethasone (Peto OR, 1.24; 95% CI, 0.79–1.95) did not. Test for subgroup differences (I^2^ = 61.9%) indicated that there was a significant difference in the serious pneumonia risk associated with different types of ICSs (Table 2 and Figure 10).

### Sensitivity Analysis

After excluding four RCTs (4,414 patients) with high risk of bias, the pooled results were similar in magnitude and direction to those (pooled results of association between various types of ICSs and pneumonia risk) obtained from all included RCTs (Table 2).

## Discussion

In this meta-analysis of 59 RCTs (including 103,477 patients), all types of ICSs, not only fluticasone, increased the pneumonia risk in COPD patients in a dose-dependent manner, and the risk was particularly evident in more severe COPD patients. Moreover, fluticasone increased the risk of serious pneumonia, while budesonide and beclomethasone did not. To our knowledge, this study was the first meta-analysis which revealed the pneumonia risk associated ICSs treatment was related with COPD severity. In addition, there was a dose-response relationship between the pneumonia risk and ICSs treatment.

At present, ICSs are widely used in the maintenance treatment of COPD patients. Since numerous COPD patients use ICSs every day, both its efficacy and safety should be considered. Although some studies have reported that fluticasone increases the pneumonia risk in COPD patients, whether other types of ICSs would increase the pneumonia risk in COPD patients remains controversial (Sin et al., 2009; Singh et al., 2009; Kew and Seniukovich, 2014; Festic et al., 2016; Yang et al., 2019; Zhang et al., 2020). In addition, it is still unclear whether different medication details and baseline characteristics (severity, age, and body mass index) of patients would affect the incidence of pneumonia after ICSs treatment.

Our results first revealed that all types of ICSs significantly increased the pneumonia risk in COPD patients regardless of treatment duration. The dose-response relationship further confirmed the causality of ICSs treatment and increased pneumonia risk in COPD patients. Moreover, our results revealed that the pneumonia risk was related with COPD severity. However, age and BMI may not be the determinants of ICSs associated pneumonia. In addition, we found that COPD patients receiving different types of ICSs may have different risk of serious pneumonia. Only fluticasone increased the risk of serious pneumonia, while other types of ICSs did not. We speculated that this may be due to the different pharmacodynamics and pharmacokinetic characteristics of different types of ICSs. Previous studies reported that fluticasone could exhibit a longer retention in the airway mucosa and thus have a more prolonged suppression of local immunity of patients (Brattsand and Miller-Larsson, 2003; Dalby et al., 2009).

### Compared With Other Studies

Several previous meta-analyses (Sin et al., 2009; Singh et al., 2009; Kew and Seniukovich, 2014; Festic et al., 2016; Yang et al., 2019; Zhang et al., 2020) also assessed the pneumonia risk associated with ICSs treatment. However, there were major differences between our meta-analysis and the previous ones in terms of selected studies, statistical analyses, and outcomes. First, we included data of some recent large-scale RCTs (Bhatt et al., 2017; Papi et al., 2017; Siler et al., 2017; Vestbo et al., 2017; Betsuyaku et al., 2018; Chapman et al., 2018; Ferguson et al., 2018a; Ferguson et al., 2018b; Frith et al., 2018; Lipson et al., 2018; Papi et al., 2018; Ichinose et al., 2019; Kerwin et al., 2019; Rabe et al., 2020) which were published after some of the previous meta-analyses. In addition, varied search strategy may be an important reason for the difference in the number of RCTs included in different meta-analyses. We systematically searched four large databases for relevant RCTs, including PubMed, Embase, Cochrane Library, and Clinical Trials.gov. In particular, we systematically searched the online supplementary documents of relevant RCTs. Indeed, pneumonia risk was not the primary outcome in most RCTs, some researchers provided data on pneumonia risk in the online supplementary documents rather than in the text. Second, compared with the previous meta-analyses, we conducted more subgroup analyses based on the baseline demographic characteristics of the patients (severity, age and BMI) to clarify possible varied pneumonia risk in different patients receiving ICSs treatment. Third, our results indicated that all types of ICSs, not only fluticasone, increase the pneumonia risk in COPD patients in a dose-dependent manner, and the risk is particularly evident in more severe patients.

In 2009, Singh et al. (Singh et al., 2009) performed a meta-analysis (18 RCTs, 16,996 patients) and concluded that ICSs (fluticasone and budesonide) treatment significantly increased the pneumonia risk in COPD patients. However, their study failed to provide some important information on ICSs associated pneumonia due to a lack of subgroup analyses based on medication details of ICSs (including dose, type and treatment duration), and subgroup analyses based on the baseline demographic characteristics of patients. In 2009, Sin et al. conducted a meta-analysis of budesonide and pneumonia risk (seven RCTs, 7,042 patients) and found that budesonide treatment for 12 mo did not increase the pneumonia risk in COPD patients. In 2014, a meta-analysis performed by Kew et al. (Kew and Seniukovich, 2014) (43 RCTs, 31,397 patients) suggested that both fluticasone and budesonide increased the serious pneumonia risk in COPD patients. However, that study did not further examine the association between other types of ICSs (momethasone and beclomethasone) and the pneumonia risk, nor conduct subgroup analyses based on the baseline demographic characteristics of patients. In 2016, another meta-analysis (29 RCTs, 33,472 patients) performed by Festic et al. (Festic et al., 2016) also revealed that ICSs increased the pneumonia risk in COPD patients. However, that study also limited by a smaller sample size and absent subgroup analyses. In addition, Yang et al. (Yang et al., 2019) conducted a meta-analysis (25 RCTs, 49,982 patients) and found ICSs significantly increased the pneumonia risk and serious pneumonia risk in COPD patients. However, their study also did not analyse the impact of baseline demographic characteristics of patients on the pneumonia risk. Moreover, in 2020, a meta-analysis (18 RCTs, 49,828 patients) performed by Zhang et al. (Zhang et al., 2020) also investigated the association between different types of ICSs and the pneumonia risk, and suggested that fluticasone increased the pneumonia risk while budesonide or beclomethasone did not. However, their results might be limited by the smaller sample size, since much fewer RCTs (especially RCTs on budesonide and beclomethasone) were included in their meta-analysis. In contrast, we searched more databases, used more search terms, and put less restrictions on literature search, which made more relevant RCTs were identified.

### Limitations and Strengths

The major strength of our study was that we conducted a comprehensive literature search including all currently available RCTs, thus ensured the generalizability of the conclusions. Moreover, the multiple subgroup analyses based on the medication details (dose and treatment duration) and baseline of patients (severity, age and BMI of patients) enhanced the reliability of the conclusions, and also provided implications for the clinical practice. As far as we know, our study is the first meta-analysis which systematically assesses the association between various types of ICSs and the pneumonia risk based on baseline characteristics of patients.

This meta-analysis had several limitations. First, none of the included RCTs were specifically designed to monitor pneumonia event, therefore, there may be underreporting of pneumonia incidence. However, the underestimate of the pneumonia risk could not substantially impact the pooled results of this meta-analysis, since underreporting of pneumonia incidence might occur equally in ICSs treatment groups and non-ICSs treatment groups. Moreover, in the sensitivity analysis, after removing four non double-blind RCTs, the results were consistent with the previous pooled results. Second, the pooled results of momethasone (four RCTs, 5,413 patients) and beclomethasone (four RCTs, 5,884 patients) may weakened by the relatively small sample size. Third, some studies were excluded because of incomplete data or non-English literature, which may lead to inevitable selection bias.

## Conclusions

ICSs treatment significantly increased the risk of pneumonia in COPD patients. There was a dose-response relationship between ICSs treatment and pneumonia risk. The pneumonia risk was related with COPD severity.

## Data Availability

All datasets generated for this study are included in the article/Supplementary Material.
