# Supplementary material for: Inhaled Corticosteroids and the Pneumonia Risk in Patients With Chronic Obstructive Pulmonary Disease: A Meta-analysis of Randomized Controlled Trials
Source: Front Pharmacol. 2021 Jun 29;12:691621. doi: 10.3389/fphar.2021.691621 (PMC8275837; doi:10.3389/fphar.2021.691621)
Supplement: Supplementary file 1 [file Table1.docx]

**Supplementary material**

Hong Chen, Jian Sun, Qiang Huang et al. Inhaled Corticosteroids and the Pneumonia Risk in Patients with Chronic Obstructive Pulmonary Disease: A Meta-analysis of Randomized Controlled Trials. Frontiers in Pharmacology.

**eTable 1: Search strategy**

| **Database** | **Search strategy** |
| --- | --- |
| Cochrane Library | #1 MeSH descriptor: [Pulmonary Disease, Chronic Obstructive] explode all trees |
|  | #2 (chronic obstructive pulmonary disease):ti,ab,kw |
|  | #3 (chronic airﬂow obstruction):ti,ab,kw |
|  | #4 (COPD):ti,ab,kw |
|  | #5 (chronic obstructive lung disease):ti,ab,kw |
|  | #6 (airﬂow obstruction, chronic):ti,ab,kw |
|  | #7 (chronic obstructive airway disease):ti,ab,kw |
|  | #8 (emphysema):ti,ab,kw |
|  | #9 (Bronchitis):ti,ab,kw |
|  | #10 #1 OR #2 OR #3 OR #4 OR #5 OR #6 OR #7 OR #8 OR #9 |
|  | #11 (ICS):ti,ab,kw |
|  | #12 (inhaled corticosteroids):ti,ab,kw |
|  | #13 (fluticasone):ti,ab,kw |
|  | #14 (flunisolide):ti,ab,kw |
|  | #15 (budesonide):ti,ab,kw |
|  | #16 (beclomethasone):ti,ab,kw |
|  | #17 (mometasone):ti,ab,kw |
|  | #18 (triamcinolone):ti,ab,kw |
|  | #19 (ciclesonide):ti,ab,kw |
|  | #20 (pulmicort):ti,ab,kw |
|  | #21 #11 OR #12 OR #13 OR #14 OR #15 OR #16 OR #17 OR #18 OR #19 OR #20 |
|  | #22 #10 AND #21 |
| Embase | #1 'chronic obstructive lung disease'/exp |
|  | #2 'chronic obstructive pulmonary disease' OR copd OR 'chronic airﬂow obstruction' OR 'chronic obstructive airway disease' OR 'emphysema' OR 'bronchitis':ab,ti |
|  | #3 #1 OR #2 |
|  | #4 ics OR 'inhaled corticosteroids' OR 'fluticasone'/exp OR fluticasone OR 'flunisolide'/exp OR flunisolide OR 'budesonide'/exp OR budesonide OR 'beclomethasone'/exp OR beclomethasone OR 'mometasone'/exp OR mometasone OR 'triamcinolone'/exp OR triamcinolone OR 'ciclesonide'/exp OR ciclesonide OR 'pulmicort'/exp OR pulmicort:ab,ti |
|  | #5 'randomized controlled trial'/exp |
|  | #6 #3 AND #4 AND #5 |
|  | #7 #6 AND [article]/lim AND [english]/lim |
| PubMed | #1 Search: ((((((((ICS[Text Word]) OR (inhaled corticosteroids[Text Word])) OR (fluticasone[Text Word])) OR (flunisolide[Text Word])) OR (budesonide[Text Word])) OR (beclomethasone[Text Word])) OR (mometasone[Text Word])) OR (triamcinolone[Text Word])) OR (pulmicort [Text Word])) OR (ciclesonide[Text Word]) |
|  | #2 Search: (((((((chronic obstructive pulmonary disease[Text Word]) OR (pulmonary disease, chronic obstructive[Text Word])) OR (COPD[Text Word])) OR (chronic airﬂow obstruction[Text Word])) OR (airﬂow obstruction, chronic[Text Word])) OR (chronic obstructive airway disease[Text Word])) OR (emphysema[Text Word])) OR (Bronchitis[Text Word]) |
|  | #3 Search: #1 AND #2 |
|  | #4 Search: #3 AND Filters: Humans |
|  | #5 Search: #4 AND Filters: Humans, English |
|  | #6 Search: #5 AND Filters: Controlled Clinical Trial, Humans, English |
|  | #7 Search: #5 AND Filters: Clinical Trial, Controlled Clinical Trial, Humans, English |
|  | #8 Search: #5 AND Filters: Clinical Trial, Controlled Clinical Trial, Randomized Controlled Trial, Humans, English |
| Clinical Trials.gov | Search term: (COPD OR chronic obstructive lung disease) AND (ICS OR inhaled corticosteroids OR fluticasone OR flunisolide OR budesonide OR beclomethasone OR mometasone OR triamcinolone OR ciclesonide OR pulmicort) |
|  | Study type: Intervention |
